# *Bifidobacterium pseudolongum *are efficient indicators of animal fecal contamination in raw milk cheese industry

**DOI:** 10.1186/1471-2180-11-178

**Published:** 2011-08-04

**Authors:** Véronique Delcenserie, Françoise Gavini, Bernard China, Georges Daube

**Affiliations:** 1 Food Sciences Department, Faculty of Veterinary Medicine, University of Liège, Sart Tilman, B43b Liege, B-4000 Belgium; 2 Technologie des Produits Animaux, Institut National de la recherche agronomique, 369 rue Jules Guesde, Villeneuve d'Ascq, F-59651 France

## Abstract

**Background:**

The contamination of raw milk cheeses (St-Marcellin and Brie) from two plants in France was studied at several steps of production (raw milk, after addition of rennet - St-Marcellin - or after second maturation - Brie -, after removal from the mold and during ripening) using bifidobacteria as indicators of fecal contamination.

**Results:**

*Bifidobacterium *semi-quantitative counts were compared using PCR-RFLP and real-time PCR. *B. pseudolongum *were detected in 77% (PCR-RFLP; 1.75 to 2.29 log cfu ml^-1^) and 68% (real-time PCR; 2.19 to 2.73 log cfu ml^-1^) of St-Marcellin samples and in 87% (PCR-RFLP; 1.17 to 2.40 log cfu ml^-1^) of Brie cheeses samples. Mean counts of *B. pseudolongum *remained stable along both processes. Two other populations of bifidobacteria were detected during the ripening stage of St-Marcellin, respectively in 61% and 18% of the samples (PCR-RFLP). The presence of these populations explains the increase in total bifidobacteria observed during ripening. Further characterization of these populations is currently under process. Forty-eight percents (St-Marcellin) and 70% (Brie) of the samples were *B. pseudolongum *positive/*E. coli *negative while only 10% (St-Marcellin) and 3% (Brie) were *B. pseudolongum *negative/*E. coli *positive.

**Conclusions:**

The increase of total bifidobacteria during ripening in Marcellin's process does not allow their use as fecal indicator. The presence of *B. pseudolongum *along the processes defined a contamination from animal origin since this species is predominant in cow dung and has never been isolated in human feces. *B. pseudolongum *was more sensitive as an indicator than *E. coli *along the two different cheese processes. *B. pseudolongum *should be used as fecal indicator rather than *E. coli *to assess the quality of raw milk and raw milk cheeses.

## Background

The genus *Bifidobacterium *represents one of the most important bacterial group in human and animal feces [1-5]. This organism has stringent nutrient requirements and grows poorly outside of the animal gut, making this bacterial group a potentially useful indicator of fecal pollution as previously described [6]. In addition, an advantage in using bifidobacteria instead of other fecal contamination indicators is the host specificity, human or animal, of some groups of *Bifidobacterium *species [3] contrary to coliforms, which are ubiquitous [7]. For example, sorbitol-fermenting bifidobacteria are associated with human fecal pollution, while *B. pseudolongum *is predominant in several animal hosts and does not have been isolated from humans [3,8,9]. *B. pseudolongum *has been isolated in more than 80% of all bifidobacteria positive fecal samples from different animals (most were collected from cattle and swine) [10]. Less than 5% of these samples were positive for bifidobacteria of human origin. This suggests that this species could be an interesting candidate for detection of animal fecal contamination.

Several studies used bifidobacteria to track fecal contamination in surface water [11-13]. Beerens and coll [14] proposed to use bifidobacteria as fecal indicators in raw milk and raw milk cheese processes and molecular method versus culture-based method have been compared for detection of bifidobacteria in raw milk [15]. A PCR method based on the *hsp60 *gene, already sequenced in most *Bifidobacterium *species [16,17] was developed for a rapid detection of bifidobacteria in a raw milk cheese process. A higher level of bifidobacteria was detected comparing to the level of *E. coli *suggesting that bifidobacteria could be a more convenient indicator. However, this method did not allow the identification of the bifidobacteria species.

Identification of *Bifidobacterium *species in highly contaminated animal feces and meat samples was studied by Gavini and coll. [10]. The use of bifidobacteria as indicator of fecal contamination along a sheep meat production chain was described by Delcenserie and coll. [18]. In that study, total bifidobacteria had been shown to be more efficient indicators than *E. coli *in carcasses samples.

Several molecular methods have been developed to detect one or several bifidobacteria species [9,12,19-22]. The purpose of most of them, however, was to detect bifidobacteria species from human origin rather than from animal origin.

In the present study, two different molecular methods were used to detect total bifidobacteria and *B. pseudolongum *present in two different French raw milk cheeses, St-Marcellin (Vercors area) and Brie (Loiret area). The results were evaluated for the potential use of bifidobacteria as indicators of fecal contamination.

## Results

### Validation of the PCR methods on pure strains

The *B. pseudolongum *(fluorochrome VIC) probe based on *hsp60 *gene was validated on 55 pure *Bifidobacterium *strains belonging to 13 different species (Table 1). The results observed with the *B. pseudolongum *probe showed a specificity of 100% and a sensitivity of 93%. Only one *B. pseudolongum *strain (LC 290/1) gave a negative result.

**Table 1:** References and source of the *Bifidobacterium *strains used for the validation of PCR assays

International or INRA internal reference	Name as received	Isolated from
ATCC 27672	*B. animalis*	Rat feces
RA20 (Biavati)	*B. animalis*	Rabbit feces
Pigeon 1/2	*B. thermophilum*	Pigeon feces
LC 458/3	*B. thermophilum*	Raw milk
B 39/3	*B. thermophilum*	Cow dung
LC 288/1	*B. thermophilum*	Raw milk
LC 110/1	*B. thermophilum*	Raw milk
T 585/1/2	*B. thermophilum*	Raw milk
Pigeon 1/1	*B. thermophilum*	Pigeon feces
T 528/4	*B. thermophilum*	Raw milk
Pigeon 4/1	*B. thermophilum*	Pigeon feces
Pigeon 4/3	*B. thermophilum*	Pigeon feces
Internal 2	B. pseudolongum **	Unknown
RU 224 (Biavati)	*B. pseudolongum *subsp. *globosum*	Bovine rumen
Internal 3	B. pseudolongum **	Unknown
MB7 (Biavati)	*B. pseudolongum *subsp. *pseudolongum*	Pig feces
LC 287/2	B. pseudolongum **	Raw milk
LC 302/2	B. pseudolongum **	Raw milk
B 81/1	B. pseudolongum **	Cow dung
LC 290/1	B. pseudolongum **	Raw milk
Poule 1/2	B. pseudolongum **	Chicken feces
LC 147/2	B. pseudolongum **	Raw milk
LC 700/2	B. pseudolongum **	Raw milk
LC 686/1	B. pseudolongum **	Raw milk
LC 680/2	B. pseudolongum **	Raw milk
LC 617/2	B. pseudolongum **	Raw milk
RU 915 B^T^	*B. merycicum*	Bovine rumen
RU 687^T^	*B. ruminantium*	Bovine rumen
LC 396/4	*B. minimum*	Raw milk
Internal 6	*B. cuniculi*	Unknown
BS3	*B. adolescentis*	Adult feces
CCUG 18363^T^	*B. adolescentis*	Adult feces
206 1a	*B. adolescentis*	Adult feces
503 1e	*B. adolescentis*	Elderly feces
1604 3a	*B. adolescentis*	Elderly feces
DSMZ 20082	*B. bifidum*	Adult feces
BS 95	*B. bifidum*	Adult feces
BS 119	*B. bifidum*	Adult feces
NCFB 2257^T^	*B. breve*	Infant intestine
Butel 10	*B. breve*	Infant feces
Butel 5	*B. breve*	Infant feces
Butel 15	*B. breve*	Infant feces
Crohn 16	*B. breve*	Adult feces
CCUG 18367^T^	*B. dentium*	Dental caries
BS 16	*B. dentium*	Adult feces
BS 39	*B. dentium*	Adult feces
BS 72	*B. dentium*	Adult feces
Crohn 24	*B. dentium*	Adult feces
NCTC 11818^T^	*B. longum*	Adult feces
BS 101	*B. longum*	Adult feces
DSMZ 20438^T^	*B. pseudocatenulatum*	Infant feces
B2b	*B. pseudocatenulatum*	Adult feces
C19i	*B. pseudocatenulatum*	Child feces
C20b	*B. pseudocatenulatum*	Child feces
C1c	*B. pseudocatenulatum*	Child feces

*: Received from B. Biavati, Instituto di Microbiologia Agaria e Tecnica, Università degli Studi di Bologna, Bologna, Italy

ATCC : American Type Culture Collection, Rockville, Maryland, USA ; CCUG : Culture Collection, University of Göteborg, Göteborg, Sweden; DSMZ : Deutsche Sammlung von Mikroorganismen und Zellkulturen GmbH, Göttingen, Germany ; NCTC : National Collection of Type Cultures, Central Public Health Laboratory, London; England); NCFB : National Collection of Food Bacteria, Shinfield, Reading, Berks, England

The PCR RFLP patterns based on 16S rDNA were validated in a previous study [20]. The RFLP patterns observed (i) with *Alu*I were named II (600-200-150-100 bp) and V (5-95-152-206-285-311), (ii) with *Taq*I were VIII (470-330-250 bp), IX (470-250-210-120 bp) and X (132-200-664). The II-VIII pattern was attributed to *B. pseudolongum *and the II-IX pattern to bifidobacteria from human origin.

### Detection of total bifidobacteria

#### - St-Marcellin process **(**Vercors's plant)

Out of the 176 analyzed samples, 153 (87%) were positive with PCR based on 16S rDNA and 154 (88%) were positive with PCR on the *hsp60 *gene (Table 2). Percentages of positive samples were very similar using one or the other method and at each studied step, from 80% (step C, after removal from the mold) to 95%, in raw milk samples. (step A).

**Table 2:** Number (percentage) of samples containing total bifidobacteria and *B. pseudolongum *in St-Marcellin and Brie processes

Process/Methods		Production steps			
**St-Marcellin**	**Total** **n = 176**	**A** **n = 44**	**B** **n = 44**	**C** **n = 44**	**D** **n = 44**

Total bifidobacteria					
PCR 16S rDNA	153 (87%)	42 (95%)	37 (84%)	35 (80%)	39 (89%)
PCR *hsp60 *gene	154 (88%)	42 (95%)	38 (86%)	35 (80%)	39 (89%)
*B. pseudolongum*					
PCR RFLP (16S rDNA)	135 (77%)/	41 (93%)/	28 (66%)/	34 (77%)/	32 (73%)/
Real time PCR (*hsp60 *gene)	120 (68%)	35 (80%)	27 (61%)	27 (61%)	31 (70%)
					

**Brie**	**Total** **n = 120**	**A'** (n = 30)	**B'** (n = 30)	**C'** **(n = 30)**	**D'** **(n = 30)**

Total bifidobacteria					
PCR 16S rDNA	107 (89%)	29 (97%)	21 (70%)	28 (93%)	29 (97%)
PCR *hsp60 *gene	105 (88%)	29 (97%)	22 (73%)	27 (90%)	27 (90%)
*B. pseudolongum*					
PCR RFLP (16S rDNA)	107 (89%)	29 (97%)	21 (70%)	28 (93%)	29 (97%)
Real time PCR (*hsp60 *gene)	ND	ND	ND	ND	ND

St-Marcellin/Production steps: A, raw milk; B, after addition of rennet; C, after removal from the mold; D, ripening (Day 21)

Brie/Production steps: A', raw milk; B', after second maturation; C', after removal from the mold; D', ripening (Day 28)

NT, not done

A significant decrease of bifidobacteria positive samples (F = 169; *P *≤ 0.01) was observed between step A (95%) and step C (80%) and a slight but not significant decrease between steps A and B and between steps B and C with both PCR on 16S rDNA gene and PCR on *hsp60 *gene methods. The lowest mean counts of bifidobacteria (Table 3), 2.34 and 2.57 log cfu g^-1 ^respectively with both methods, were found at step C (after removal from the mold). Next, surprisingly, a significant increase of these counts was observed during ripening (F values of 14.16 and 49 respectively; *P *≤ 0.01) to reach means as high as 3.71 and 3.88 log cfu g^-1 ^at step D with the two respective PCR methods.

**Table 3:** Mean counts (log cfu ml^-^^1 ^or g^-^^1^) of total bifidobacteria, *B. pseudolongum *and *E. coli *in St-Marcellin and Brie processes

Process/Species	Method	Production step *			
**St-Marcellin**		**A**	**B**	**C**	**D**

Total bifidobacteria	PCR 16SrDNA	3.05 ± 1.29/	2.85 ± 1.25/	2.34 ± 1.48/	3.71 ± 1.89/
	PCR hsp60 gene	3.03 ± 2.26	3.03 ± 2.15	2.57 ± 2.25	3.88 ± 1.97
*B. pseudolongum*	PCR-RFLP (16S rDNA)	2.29 ± 1.24/	1.75 ± 1.43/	2.23 ± 1.46/	1.88 ± 1.40/
	Real time PCR (hsp60 gene)	2.73 ± 2.30	2.29 ± 2.18	2.19 ± 2.11	2.48 ± 2.17
*E. coli*	Culture	1.03 ± 1.31	1.29 ± 1.25	0.51 ± 0.93	0.25 ± 0.63

**Brie**		**A'**	**B'**	**C'**	**D'**

**Total bifidobacteria**	PCR 16SrDNA	2.13 ± 0.73/	1.17 ± 0.91/	2.40 ± 1.16/	2.37 ± 0.81/
	PCR hsp60 gene	2.03 ± 0.85	1.23 ± 1.04	2.20 ± 1.13	1.90 ± 0.92
*B.pseudolongum*	PCR-RFLP (16S rDNA)	2.13 ± 0.73/	1.17 ± 0.91/	2.40 ± 1.16/	2.37 ± 0.81/
	Real time PCR (hsp60 gene)	ND	ND	ND	ND
*E. coli*	Culture	0.00 ± 0.00	0.14 ± 0.41	2.49 ± 0.71	1.65 ± 0.91

St-Marcellin/Production steps: A, raw milk; B, after addition of rennet; C, after removal from the mold; D, ripening (Day 21)

Brie/Production steps: A', raw milk; B', after second maturation; C', after removal from the mold; D', ripening (Day 28)

ND, not done

#### - Brie process (Loiret's plant)

Out of the 120 analyzed samples, 107 were positive (89%) with PCR based on 16S rDNA gene and 105 (88%) with PCR on *hsp60 *gene for total bifidobacteria (Table 2). These percentages were very close to those found along the St-Marcellin process.

The lowest mean counts of bifidobacteria (Table 3) were found at step B' (after second maturation), 1.17 and 1.23 log cfu g^-1 ^respectively with PCR based on 16S rDNA gene and PCR on *hsp60 *gene. The highest mean counts were found at step C' (after removal of the mold), 2.4 and 2.2 log cfu g^-1 ^for PCR on 16S rDNA gene and PCR on *hsp60 *gene.

No differences were observed in total bifidobacteria level along the production chain, from 2.13 log cfu ml^-1 ^at step A' to 2.20 log cfu g^-1 ^at step C' and 1.90 log cfu g^-1 ^at step D' excepted for a marked decrease observed at step B', after the second maturation (1.17 log cfu g^-1^; F = 10.6; *P *< 0.01). At the step B', the temperature had been increased from 10-12°C (cold maturation) to 34°C-36°C (hot maturation). Before the molding step (still between 34°C and 36°C), the bifidobacteria level increased again (results not shown). The decrease of bifidobacteria cannot be explained by the temperature or pH (around 6.5), because these parameters did not change at these steps. A more probable explanation could be the addition of starters, leading to competition between microbial species.

### Detection of B. peudolongum and E. coli

#### - St-Marcellin process (Vercor's plant)

Out of the 176 samples analyzed by PCR-RFLP, 135 (77%) were II-VIII type positive (*B. pseudolongum*), *B. pseudolongum *was found in at least 66% of (step B) to 93% of (step A) samples (Table 2).

Using real-time PCR (Table 2), out of the 176 analyzed samples, 120 samples (68%) were positive with the *B. pseudolongum *probe, a little bit less than the number found using PCR-RFLP (77%).

No significant difference was observed between the *B. pseudolongum *counts at the different steps.

In addition, three more combined patterns were observed along the cheese process: II-IX (presumed human origin bifidobacteria [23], V-IX and V-X. One hundred and eight samples (61%) were V-X type positive and 31 (18%) were V-IX type positive. Only 3 samples (1.5%) were II-IX type positive.

It was not possible to attribute the profile combinations V-X and V-IX to a known species of bifidobacteria from our pure strains collection (Table 1). These two populations were further investigated and the preliminary results indicate that they belong respectively to the recently described species *B. crudilactis *and *B. mongoliense *(results not shown).

A high number of *E. coli *negative samples (101/160; Table 4) were observed: 48% of them were *B. pseudolongum *positive. The highest percentage of negative samples (83%) was found at step D, during ripening. Mean counts of *E. coli *(Table 3) were very low at steps C and D (0.51 and 0.25 log cfu g^-1 ^respectively) because of the high numbers of negative samples observed at these steps. For statistical calculations, values of 1 log below the detection limit were attributed to negative *E. coli *samples. For example, values of 1 CFU g^-1 ^were attributed to negative samples from step A' and B', 10 CFU g^-1 ^to negative samples from step D' and 100 CFU g^-1 ^to negative samples from step C'. Indeed, samples from step A' and B' (cold and hot maturation) were analyzed from pure dilution, while samples from step C' (after removing from the mold) and D' (ripening) were respectively analyzed from 10^-3 ^and 10^-2 ^dilutions.

**Table 4:** Number (percentage) of samples positive for *B. pseudolongum *and/or *E. coli *in St-Marcellin and Brie processes

		Production steps			
**St-Marcellin**	**Total**	**A**	**B**	**C**	**D**
	**n = 160**	**n = 40**	**n = 36**	**n = 42**	**n = 42**

BP+/E+	43 (27%)	18 (45%)	15 (42%)	5 (12%)	5 (12%)
BP+/E-	77 (48%)	18 (45%)	12 (33%)	22 (52%)	26 (62%)
BP-/E+	16 (10%)	1 (2.5%)	6 (17%)	7 (17%)	2 (5%)
BP-/E-	24 (15%)	3 (7.5%)	3 (8%)	8 (19%)	9 (21%)

**Brie**	**Total**	**A'**	**B'**	**C'**	**D'**
	**n = 118**	**n = 30**	**n = 28**	**n = 30**	**n = 30**

BP+/E+	22 (19%)	0	1 (4%)	8 (27%)	13 (43%)
BP+/E-	83 (70%)	29 (97%)	18 (64%)	20 (67%)	16 (53%)
BP-/E+	3 (3%)	0	1 (4%)	2 (7%)	0
BP-/E-	10 (8%)	1 (3%)	8 (29%)	0	1 (3%)

BP : *B. pseudolongum *; E : *E. coli*

St-Marcellin/Production steps: A, raw milk; B, after addition of rennet; C, after removal from the mold; D, ripening (D 21)

Brie/Production steps: A', raw milk; B', after second maturation; C', after removal from the mold; D', ripening (D 28)

#### - Brie process (Loiret's plant)

Out of the 120 samples analyzed by PCR-RFLP, 107 (89%) were II-VIII type positive (*B. pseudolongum*), corresponding to the percentage of samples containing total bifidobacteria (Table 2).

The number of *E. coli *negative samples was also very high (93/118; Table 4); among them, 89% were *B. pseudolongum *positive/*E. coli *negative. In addition, an increase of *E. coli *counts was observed during stages C' and D' (removing from the mold and ripening) with values of respectively 2.5 and 1.7 log cfu g^-1^.

## Discussion

### Use of *B. pseudolongum *as a fecal indicator rather than total bifidobacteria

Bifidobacteria contaminated 88% of the studied samples in both cheese processes. It was not surprising to detect *B. pseudolongum *in 68% of the samples from Vercors's plant and in 87% of the samples from Loiret's plant. Indeed, this species was also the most frequently isolated species in raw milk samples on farms [14], which were contaminated by cow dung. *B. pseudolongum *was present in 97% of cow dung samples [14] and was also the most frequent species in other animal feces on the farm [10].

In one of the plants (Vercors, St-Marcellin process), the mean counts of bifidobacteria (3.88 log cfu ml^-1^) were higher than those of *B. pseudolongum *(2.48 log cfu ml^-1^) at step D, during ripening.

This suggests that other bifidobacteria species than *B. pseudolongum *are present in these samples as suspected by the presence of other PCR RFLP patterns than the one of *B. pseudolongum*. Their origin is unknown. These bacteria need to be further studied. Therefore *B. pseudolongum *is a better candidate as fecal indicator than total bifidobacteria. It is present along the two processes and remains significantly stable. In addition, its animal origin gives origin of the contamination.

No significant difference was observed between *B. pseudolongum *semi-quantitative counts with PCR-RFLP or real-time PCR at each step of production. The PCR-RFLP method was slightly more sensitive with 77% of positive sample against 68% for real-time PCR. This difference is explained by false negative observed with real-time PCR at lower dilutions. Those false negative can be due to PCR inhibition. The development of an internal control for the real-time PCR as the one developed for the PCR-RFLP could help to control this phenomenon in the future. Both methods can be applied in routine analysis. However, real-time PCR is faster and less labor consuming than PCR-RFLP. This method seems to be the method of choice in this kind of application.

### Use of *B. pseudolongum *as fecal indicator rather than *E. coli*

The high percentage of *B. pseudolongum *positive - *E. coli *negative samples (Table 4) supports the proposition to use *B. pseudolongum *as indicator of fecal contamination rather than *E. coli *in raw milk cheese samples. Forty-eight percent and 70% respectively of St-Marcellin and Brie samples were *B. pseudolongum *positive and *E. coli *negative while only 10% and 3% were *B. pseudolongum *negative and *E. coli *positive. *E. coli *was absent in numerous samples during ripening in St-Marcellin process or at maturation step in Brie process.

The comparison between mean counts of *E. coli *and *B. pseudolongum *showed that *B. pseudolongum *counts were always higher than those of *E. coli *in the two plants (Table 3). These differences were highly significant at steps A, C and D (F = 20.97; 43.18 and 48.37 respectively; *P *< 0.0005) in the St-Marcellin's process, at steps A', B' and D' (F = 326; 37; *P *< 0.0005 and F = 11.3; *P *< 0.01, respectively) in Brie's process. In addition, *E. coli *counts were not stable during both processes with either an increase (at removal from the mold step of Brie's process) or a decrease (ripening or maturation step of both processes). Reduction and even disappearance of *E. coli *during ripening in St-Marcellin's process or during maturation step in Brie's process could be due to low pH and to inhibition by competitive flora as it was shown by Caridi and coll. [24,25].

These observations confirmed the fact that *E. coli *is not a suitable fecal indicator for both of these processes. In both processes, absence of *E. coli *did not mean absence of fecal contamination, whereas presence of *B. pseudolongum *pointed out a very large fecal contamination from animal origin.

Up to our knowledge and till now, the species *B. pseudolongum*, from animal origin, is not used as a probiotic in human food. However, it is important to point out that those results shown in relation to raw milk cheese must not be generalized for other milk products such as fermented milk containing probiotics. In those products, the presence of specific strains of bifidobacteria is a desired quality criterion.

## Conclusion

Feces from animal origin appears to be the most probable external source of contamination by *B. pseudolongum *of the raw milk used along the two raw milk cheese processes under study. This species contaminates all steps of the processes.

*B. pseudolongum *is the most frequent species in animal feces [10,14,18]. Then it could be chosen as an efficient indicator of fecal contamination as it remained stable along the processes with semi-quantitative mean counts equal or close to 10^3 ^cfu ml^-1 ^or g^-1^. Presence of an increase of total bifidobacteria during ripening in Marcellin's process does not allow using total bifidobacteria as fecal indicator. In addition, the reason for that increase is not known yet. Eventually, another reason to use *B. pseudolongum *as indicator is the high number of *E. coli *negative samples. This confirms interest in using this species rather than *E. coli.*

Results were very similar with both PCR-RFLP and real-time PCR in the St-Marcellin process. Both methods can be applied in routine analysis. However, PCR-RFLP is less practicable and less fast than real-time PCR. Real-time PCR seems to be the method of choice in this kind of application where rapidity and easiness are important. Further improvements such as addition of an internal control to detect PCR inhibition needs to be done. It could then lead to the successful use of bifidobacteria as fecal indicators by detecting and quantifying *B. pseudolongum *at different steps and at the end of raw milk cheese production chains. *B. pseudolongum *detection or quantification could also be used for raw milk quality assessment in the plant. Other fecal bacteria such as enterococci could have been considered as well as authenticity markers as they are predominant in raw milk. However, enterococci can survive to pasteurization and thermization processes [26,27]. This disqualifies them as "raw milk" authenticity markers. In addition, another advantage of *B. pseudolongum *is to be of strict fecal animal origin and unable to multiply during the manufacturing process, contrarily to other fecal bacteria potentially present in raw milk.

The increase in total bifidobacteria counts during ripening in the St-Marcellin process was partially explained by the presence of *B. crudilactis *strains, a recently described species [28]. Future work is currently done to study the interactions of strains belonging to this species and to a newly described one, *B. mongoliense *[29], in the raw milk cheese production chains.

## Methods

### Target DNA preparation from pure strains

Fifty-five reference strains belonging to 13 *Bifidobacterium *species (Table 1) were used in this study. Seven species were from human origin, while six others were from animal origin. The *Bifidobacterium *strains were subcultured in Brain Heart Infusion (BioRad, Marnes-la-Coquette, France) at 37°C for 48 to 72 h under anaerobic conditions and DNA was extracted as described previously [15].

### Target DNA preparation from raw milk cheese samples

#### - Raw milk cheese processes

##### Vercors's plant (Table 5)

**Table 5:** pH and temperature at the different production steps in L'Etoile du Vercors (St-Marcellin)

Production steps (Analysed step)	pH	Temperature
**Milk at the factory (A)**	6.7	4°C
After maturation (2h30)	6.5	22°C
**After rennet/Day 0 (B)**	6.45	22°C
After moulding/Day 1	4.3	22°C
**After removal from the mould/Day2 (C)**	4.35	22°C
Ripening/Day 15	4.7	12°C (from J+8)
**Ripening/Day 21 (D)**	5.5	12°C
Ripening/Day 28	>6	12°C

In the first plant under study from the Vercors area in France (St-Marcellin cheese), milk was collected on farms and stored in tanks at the plant at 4°C as already described [15]. Milk was prepared for maturation by addition of cream, starter and surface flora. Temperature was increased to 22°C. Animal rennet was added (Day 0).

On the next day (Day 1), the following steps were successively performed: molding, a first manual turnover, a manual salting and a second turnover. During that day, pH decreased from 6.5 to 4.3 while temperature remained stable (22°C). On the second day, cheeses were removed from the molds and a new manual or mechanical salting was performed. Ripening was then carried out for 28 days. Temperature was 12°C from Day 8. During that stage, pH slowly increased from 4.35 (at the beginning of ripening), to 4.7 (Day 15), to 5.5 (Day 21), to more than 6 (Day 28).

Forty-four raw milk cheeses at 4 different steps (176 samples) were analyzed at the following production steps: raw milk (Step A, Day 0), after addition of rennet (Step B, Day 0), after removal from the mold (Step C, Day 2) and during ripening (Step D, Day 21).

##### Loiret's plant (Table 6)

**Table 6:** pH and temperature at the different production steps in Les Courtenay (Brie)

Production steps	pH	Temperature
**Milk at the factory (A')**	6.7 - 6.90	<6°C
After the 1 ^st ^maturation (cold)	6.65 - 6.75	10 to 12 °C
**After the 2 **^**nd **^**maturation (hot) (B')**	6.30 - 6.50	34 to 36°C
After curdling	6.25 - 6.35	34 to 36°C
**After removal from the mould (C')**	4.70 - 5.00	20 to 22°C
After salting (side 2)	4.70 - 5.00	17 to 20°C
**Ripening (Day 28) (D')**	5.00 - 5.60	6 to 10°C
Ripening (Day 45)	6.50 - 7.00	6 to 10°C

In the second plant under study from Loiret area in France (Brie cheese), milk was collected on farm and stored at a temperature below 6°C to allow decantation and standardization of the cream. After two different maturation steps: cold (10 to 12°C, 16 to 24 h) and hot (34 to 36°C, 15 to 40 h), rennet was added, a manual molding was performed and followed by two turnovers (10 h and 14 h after molding). The starter was also added just after the cold maturation. Then, cheeses were removed from the molds and salted on each side. Several hours later, after mold inoculation of cheeses, drying was performed for 2 to 6 days. Finally, ripening had been allowed for a period of about 3 weeks.

Thirty raw milk cheeses were analyzed at four different production steps (120 samples): raw milk (Step A', Day 0), after the second maturation (Step B', between Day 1 and Day 3), after removal from the mold (Step C', Day 3) and during ripening (Step D', Day 28).

#### - Enrichment step

The enrichment medium was Brain Heart Infusion (BHI, 37 g l^-1^, Bio-Rad, Marnes-la-Coquette, France), supplemented with several components (propionic acid, 5 ml l^-1^; Fe-citrate, 0.5 g l^-1^; cystein chlorhydrate, 0.5 g l^-1^; yeast extract, 5 g l^-1^; agar, 2 g l^-1^) and mupirocin (Lithium mupirocin, GlaxoSmithKline, England) as the selective agent at a final concentration of 80 mg l^-1 ^[23].

One ml of milk or 1 g of raw milk cheese was transferred into a tube of enrichment medium and 1 ml of each of the ten fold appropriate sample dilutions in quarter-strength Ringer solution containing cystein chlorhydrate (0.3 g l^-1^) was also inoculated in tubes of enrichment medium in order to detect bifidobacteria in milk and raw milk cheese until the 10^-6 ^dilution. Estimated mean counts of bifidobacteria were obtained using the last positive dilution.

Tubes were incubated at 37°C for 72 h in aerobiosis, as the bacteria were able to grow in depth because of the presence of agar in the medium.

#### - DNA extraction

DNA was extracted from culture broths obtained after the enrichment step (from non-diluted to 10^-6 ^dilution). One ml of each homogenized content from each dilution was transferred in a microcentrifuge tube and centrifuged at 12,000 × *g *for 2 min using a bench-top centrifuge. The pellets were transferred into 1 ml of sterile molecular grade water. The DNA was extracted using the Wizard Genomic DNA purification kit (Promega, Madisson, WI, USA) with addition of lysozyme (10 mg/ml, Eurogentec, Seraing, Belgium), as recommended for Gram-positive bacteria. DNA samples were analyzed pure or 10 fold-diluted in case of PCR inhibition.

### Molecular protocols for bifidobacteria detection

#### PCR-RFLP protocol based on the 16S rDNA gene (PCR-RFLP)

The PCR method for the detection of the *Bifidobacterium *genus consisted of primers targeting the 16SrDNA gene followed by a digestion using 2 restriction enzymes for species detection. A 1050 bp amplicon of the 16S rDNA gene was generated using primers: 16S up: 5'-AAT AGC TCC TGG AAA CGG GT-3' and 16S down: 5'-CGT AAG GGG CAT GAT GAT CT-3' (Eurogentec, Seraing, Belgium; Genbank PUID: updown16S EOY_1) as previously described [23]. The digestion of the PCR products for species detection was performed using two enzymes: *Alu*I and *Taq*I (Roche; Basel, Switzerland) as described previously [23]. Following the digestion, the products were analyzed by gel electrophoresis using 2.5% agarose gel. The profiles were analyzed using the Kodak 1D software (Thermolabsystems, Brussels, Belgium).

#### Real-time PCR protocol based on the hsp60 gene

A first step consisted in PCR targeting the *hsp60 *gene for detection of positive samples for bifidobacteria. Next, real-time PCR was applied to positive samples for species identification.

The PCR procedure for detection of the *Bifidobacterium *genus was described in a previous study [15]. The following primers were used: B11 up: 5'-GTS CAY GAR GGY CTS AAG AA-3' and B12 down: 5'-CCR TCC TGG CCR ACC TTG T-3' (Sigma Genosys, UK; Genbank PUID: hsp60updown EOY_2), to obtain a 217 bp amplicon of the *hsp60 *gene. An internal DNA control was included in each reaction. The products were analyzed by gel electrophoresis using 1.5% agarose gels.

Species detection was carried out by real-time PCR using TaqMan technology. The degenerated primers specific to the *Bifidobacterium *genus were the same than those utilized for the PCR on the *hsp60 *gene. One probe was chosen from *hsp60 *sequences of *B. pseudolongum *after *hsp60 *gene sequencing of 40 bifidobacteria strains: 3 *B. adolescentis*, 3 *B. pseudocatenulatum*, 2 *B. breve*, 2. *B. longum*, 2 *B. bifidum*, 14 *B. pseudolongum *and 10 *B. thermophilum *(data not shown). The bifidobacteria sequences were aligned using the program ClustalW from the European Bioinformatics Institute (http://www.ebi.ac.uk/clustalw/). The alignments revealed specific sequences for *B. pseudolongum*. From these sequences, the probe was designed using the primers and probes design guidelines provided by Applied Biosystems (Applied Biosystems, Foster city, USA). To check for specificity, the selected probes were compared to all available *hsp60 *gene sequences using the BLAST database search program (http://www.ncbi.nlm.nih.gov/BLAST/). The *B. pseudolongum *probe was VIC- CTCCGACGCGATCGT-DQ (Applied Biosystems, Foster city, USA; Genbank PUID: TaqManPseudolongum EOY_3).

Amplification reaction mixtures contained between 10 to 50 ng of DNA, 12.5 ml of qPCR tm Mastermix (Eurogentec, Seraing, Belgium), 960 nM of each primer, 50 to 150 nM of fluorogenic probe, and 5 mM MgCl_2 _in a total volume of 25 μl. In each microwell plate, one well was used as non-template control, which contained all the reagents except the DNA sample. The amplification, 50°C for 2 min, 95°C for 10 min, and then 40 cycles of two-temperature PCR (95°C for 30 s and 60°C for 90 s) and detection was carried out on an ABI Prism 7000 sequence detection system (Applied Biosystems, Foster city, USA). The PCR results for the samples were expressed as delta Rn (relative sensitivity) fluorescence signal. A sample was considered as positive when the relative fluorescence value was higher than 500.

The degenerated pair of primers specific to the *Bifidobacterium *genus was tested for its specificity in a previous study [15]. To check specificity of the probe, a real-time PCR was performed on 55 strains belonging to 13 different *Bifidobacterium *species (Table 1). The limit of detection was of minimum 10 ng of DNA/reaction.

### *E. coli *detection

*E. coli *were enumerated by culture method on the Coli ID medium (BioMerieux, France; [30]).

### Statistical analysis

The Mc Nemar test was used to evaluate statistical significance of the data. All dilutions were tested as separate values. To see if results obtained at different steps of the raw milk cheese production were significantly different, an ANOVA test was performed.

## Competing interests

The authors declare that they have no competing interests.

## Authors' contributions

VD carried out the molecular experiments and drafted the manuscript. FG carried out the cultural methods experiments, participated in the design and coordination of the study and helped to draft the manuscript. BC helped in the design of the molecular experiments. GD participated in the design of the study and helped to draft the manuscript. All authors read and approved the final manuscript.
